# Elimination of tumorigenic pluripotent stem cells from their differentiated cell therapy products: An important step toward ensuring safe cell therapy

**DOI:** 10.1016/j.stemcr.2025.102543

**Published:** 2025-06-19

**Authors:** Afsaneh Yazdani Movahed, Rana Bagheri, Pierre Savatier, Tomo Šarić, Sharif Moradi

**Affiliations:** 1 Department of Stem Cells and Developmental Biology, Cell Science Research Center, Royan Institute for Stem Cell Biology and Technology, ACECR, Tehran, Iran; 2 University Lyon, University Lyon 1, INSERM, Stem Cell and Brain Research Institute U1208, 69500 Bron, France; 3 Center for Physiology and Pathophysiology, Institute for Neurophysiology, Faculty of Medicine and University Hospital Cologne, University of Cologne, Cologne, Germany

**Keywords:** pluripotency, teratoma, teratogenesis, cell therapy, miRNA, transplantation, cell graft

## Abstract

Human pluripotent stem cells (hPSCs) are considered a promising tool for regenerative medicine due to their unique self-renewal and multi-lineage differentiation capabilities. Although over 100 clinical trials have employed hPSC-derived products to treat life-threatening diseases, the tumorigenic risk posed by residual undifferentiated hPSCs remains a formidable obstacle to their clinical implementation. In this review, we summarize current strategies to eliminate tumorigenic hPSCs, most of which target hPSC-specific markers, and critically evaluate the advantages and limitations of each approach. Finally, we discuss various methods that can be used to evaluate the efficiency of pluripotent stem cell (PSC) elimination.

## Graphical abstract

**Figure:**
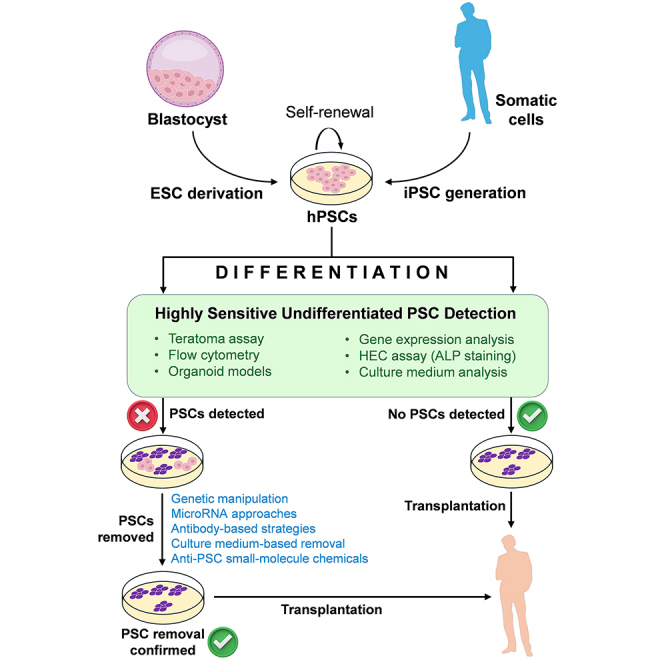

## Teaser

Moradi and colleagues review strategies to eliminate tumorigenic undifferentiated hPSCs from therapeutic cell populations. They highlight various strategies, including small molecules, microRNAs, and antibody-based approaches, as clinically viable solutions, addressing critical safety barriers in hPSC-based regenerative medicine. The study underscores the need for robust, safe, and scalable methods to ensure tumor-free PSC-based cell therapies.

## Introduction

**Figure 1:**
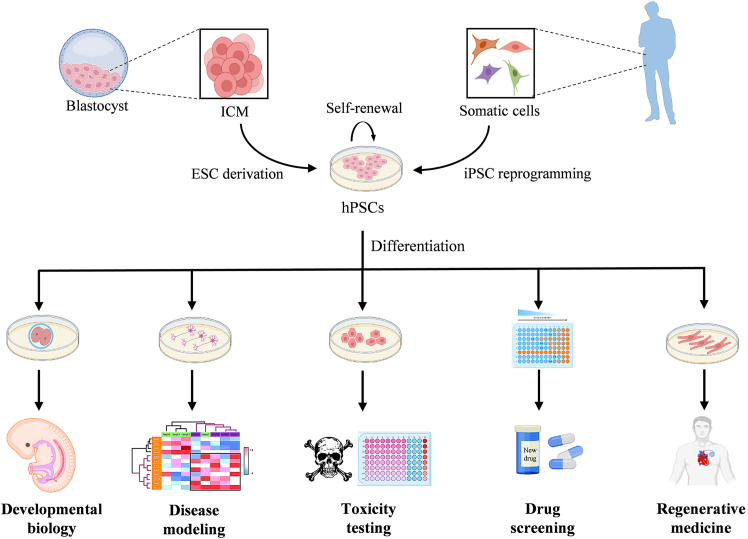
Biomedical applications of hPSCs Human ESCs and iPSCs have the ability to self-renew indefinitely and differentiate into all cell types of the body. Consequently, they can serve as an inexhaustible source of differentiated cells for various biomedical applications, including the investigation of human embryonic development, drug screening, toxicity testing, disease modeling, and regenerative medicine.

Human pluripotent stem cells (hPSCs) are most commonly derived either from the inner cell mass of blastocyst-stage embryos, known as embryonic stem cells (hESCs), or from somatic cells through epigenetic reprogramming, known as induced pluripotent stem cells (hiPSCs). These cells possess unique features, including unlimited self-renewal and the ability to differentiate into derivatives of all three primary germ layers (Moradi et al., 2019; Ramalho-Santos and Willenbring, 2007). These characteristics provide a replenishable source of various tissue-specific cell types, making hPSCs invaluable for regenerative medicine, disease modeling, toxicity testing, and drug discovery (Figure 1). Since 2011, up to 109 clinical trials using hPSC derivatives have been initiated to treat a wide range of incurable and life-threatening diseases. Although hESC derivatives are still used in about 50% of ongoing trials, nearly twice as many trials have been initiated with hiPSC derivatives compared to hESC derivatives in the past 5 years (Kobold et al., 2023). Among the therapeutic cell types employed in these clinical studies, the most frequently used are the hPSC-derived retinal pigment epithelial cells (22% of all studies), followed by natural killer (NK) cells (18%), mesenchymal stromal cells (MSCs, 12%), various types of neural cells (14%), cardiac muscle cells (12%), and endocrine pancreatic cells (8%) that are being explored for treating conditions such as macular degeneration, cancer, spinal cord injury, Parkinson’s disease, heart failure, and diabetes mellitus (Kobold et al., 2023). Early-phase results from most of these trials indicate that the therapies are generally well tolerated and safe. While therapeutic benefits have been reported in some studies on human subjects (Christiansen and Kirkeby, 2024; Wang et al., 2024), for most indications, efficacy reported in animal models still needs to be demonstrated in humans (Ilic and Ogilvie, 2022; Kirkeby et al., 2023; Kobold et al., 2023).

Cell-based tissue repair is a rapidly evolving field with the potential to transform the future of medicine, yet it still faces significant technological and regulatory challenges (Christiansen and Kirkeby, 2024; Jha et al., 2021; Madrid et al., 2024). Notably, the same cellular features that make pluripotent stem cells (PSCs) valuable for biomedical applications can also lead to tumor formation, specifically teratomas (from the Greek word “teratos,” meaning monster), tumors that form when undifferentiated stem cells are transplanted directly into various anatomical sites of syngeneic or immunodeficient recipients. A teratoma is a benign tumor composed of randomly distributed differentiated structures that resemble various adult tissues, arising from all three germ layers (Blum and Benvenisty, 2008; Lee et al., 2013a). Stem cell-derived teratomas form when mammalian embryos, embryoid body-derived cells, or undifferentiated PSCs are transplanted (Gutierrez-Aranda et al., 2010; Solter et al., 1981), posing a critical safety challenge to stem cell therapy. Inducing PSCs to differentiate into specific cell types leads to the loss of pluripotency in undifferentiated PSCs and dramatically reduces the likelihood of tumor formation.

However, it has been shown that even a few remaining undifferentiated PSCs in the population of differentiated cells can result in teratoma formation following transplantation (Lawrenz et al., 2004; Parr et al., 2016). Therefore, to minimize the risk of tumorigenicity, it is crucial to ensure the complete elimination of all undifferentiated PSCs intended for therapy, highlighting the need for highly efficient differentiation protocols.

Despite prolonged differentiation procedures, some undifferentiated PSCs may remain at the end of a protocol (Cui et al., 2013; Hentze et al., 2009; Roy et al., 2006), and transplantation of differentiated cells derived from both mouse and human PSCs can still lead to the formation of tumors or tumor-like structures, including teratomas (Cui et al., 2013; Doi et al., 2012; El Khatib et al., 2016; Germain et al., 2012; Lee et al., 2013a), or tumors representative of differentiated lineages (Nori et al., 2015). For example, Nori and colleagues demonstrated that small tumors capable of significantly impairing organ function can form from hiPSC-derived neurospheres. These tumors proliferated and differentiated *in vivo* into various neural lineages and undifferentiated nestin-positive neural cells after long-term observation (Nori et al., 2015). This finding suggests that tumors may develop not only from undifferentiated PSCs contaminating therapeutic cell populations but also through the reactivation of tumorigenic transgenes, such as *OCT4*, or activation of endogenous oncogenes in differentiated PSC precursor cells. Thus, using genetically intact integration-free induced pluripotent stem cells (iPSCs) for transplantation is essential to avoid transgene-induced tumorigenesis.

The importance of strictly monitoring the purity of hiPSC-based products and ensuring that they do not contain tumorigenic cells is underscored by a recent clinical case report describing the occurrence of an immature teratoma in a patient who received an intramuscular injection of autologous iPSC-derived pancreatic beta cells for the treatment of diabetes (Han et al., 2022). The tumor was detected at the injection site 2 months after transplantation and had already metastasized to the regional lymph nodes. It contained OCT4- and SOX2-positive cells, grew rapidly, showed strong vascularization, and was unresponsive to chemotherapy recommended for immature teratomas. This case highlights the need to develop and standardize efficient hPSC removal strategies and implement strict quality control measures for all hiPSC-based therapies.

Moreover, the genetic instability of PSCs poses another major concern because chromosomal abnormalities and sub-karyotypic alterations acquired during reprogramming, *in vitro* maintenance, or differentiation could increase the tumorigenic potential of the cells. Such genetic aberrations can activate oncogenes, such as *MYC*, and deactivate tumor suppressor genes, such as *P53*, thus elevating the risk of tumorigenicity. Prolonged culture of PSCs frequently results in the accumulation of genetic alterations, such as chromosomal aberrations (Assou et al., 2020), copy-number variations, and point mutations (Baker et al., 2016; Fu et al., 2021). The most frequently observed genetic abnormalities in hPSCs are trisomy of chromosome 20, trisomy of 12q, and gains of partial or entire chromosomes 1, 17, and X (Andrews et al., 2022; Assou et al., 2020). These genetic instabilities may carry over into differentiated cells or could occur *de novo* during differentiation, posing a significant risk for the safe application of PSC-derived therapies (Andrews et al., 2022). Therefore, it is imperative to address these issues to mitigate oncogenic risks in regenerative medicine.

Given that the risk of tumor formation associated with hPSC-based cell therapy represents a rare but robust threat to the safety of such therapies, several strategies have been proposed to remove residual undifferentiated hPSCs. These strategies include genetic manipulations, immunological targeting, and the use of pharmacological small-molecule compounds (Ben-David and Benvenisty, 2014; Jeong et al., 2017; Wuputra et al., 2020), each with its own advantages and disadvantages. As the use of hPSCs in regenerative medicine rapidly expands, adopting the safest and most efficient methods for hPSC removal becomes critically important. In this review, we summarize the various strategies for eliminating undifferentiated hPSCs from differentiated cell populations intended for cell therapy. We also explore the advantages and disadvantages of each approach and present key questions and recommendations to maximize the safety of hPSC-based cell therapy by minimizing the risk of teratoma formation. Lastly, we review methods that can be used to assess the efficiency of PSC removal.

## Tumorigenicity as a major clinical hurdle in PSC-based cell therapy

PSC-based cell therapies face several clinical obstacles, including incomplete differentiation into mature functional cells, low survival rate, poor integration of transplanted cells, the risk of immune rejection, and concerns about their long-term safety, particularly the risk of tumorigenesis (Bragança et al., 2019; Nagoshi et al., 2019; Romito and Cobellis, 2016; Trounson and McDonald, 2015; Tsuji et al., 2019). For instance, studies have shown that in mouse embryonic stem cells (ESCs), the presence of only 20 to 100 undifferentiated ESCs within a population of differentiated cells could eventually lead to teratoma formation (Lawrenz et al., 2004). However, it is important to note that these studies were conducted using murine ESCs in a mouse model, and the findings may not be directly applicable to hPSCs. Furthermore, when injected into immunodeficient mouse models, hPSCs are less tumorigenic than murine cells, requiring more cells to induce tumors and taking longer to develop tumors after injection. The tumorigenicity of hPSC transplantation in human recipients remains unknown. Nevertheless, these studies highlight the inherent risk of tumorigenesis associated with PSCs, even after *in vitro* differentiation.

The teratoma risk associated with hiPSCs has been assessed following intravenous injection of cells expressing luciferase into immunodeficient NSG mice (Bedel et al., 2017). This study found that 2 × 10^5^ iPSCs were sufficient to induce teratoma growth, with tumors developing in multiple organs several weeks post transplantation, as expected following systemic cell administration. In contrast, Gropp and colleagues demonstrated reproducible teratoma formation in all immunodeficient NOD/SCID mice after subcutaneous co-transplantation of 1 × 10^5^ undifferentiated hESCs with mitotically inactivated feeder cells (human foreskin fibroblasts) and Matrigel (Gropp et al., 2012). Under these conditions, teratomas also developed after transplantation of only 100 hESCs, but this occurred in just 2 of 30 transplanted animals (6.7%) and required a longer follow-up period (10–12 weeks) compared to animals that received 1 × 10^5^ hESCs (2–4 weeks). The efficiency of PSC differentiation protocols varies, and using the most effective protocols reduces the number of undifferentiated PSCs in the differentiated cell population, thereby decreasing the likelihood of tumor formation (Doi et al., 2012; Kriks et al., 2011).

PSCs may also give rise to malignant tumors known as teratocarcinomas, which contain both a differentiated part resembling teratomas and an undifferentiated part comprising embryonal carcinoma cells (ECCs), which are undifferentiated PSCs imparting malignant and invasive characteristics to the tumor (Chambers and Smith, 2004; Gordeeva and Nikonova, 2013; Martin and Evans, 1975). However, using a BrdU incorporation assay, Blum and Benvenisty demonstrated that hESC-derived teratomas induced in immunodeficient mice contain highly dividing cells scattered throughout the tumor (Blum and Benvenisty, 2007). Interestingly, BrdU incorporation was detected only in differentiated cells, with no evidence of undifferentiated hESCs in any tumor area. Gertow and co-workers also failed to detect cells expressing typical markers for undifferentiated hESCs or malignant cells in any of the tumor samples examined, despite extensive mitotic activity throughout the tumor (Gertow et al., 2004). This contrasts with teratocarcinomas formed from mouse ESCs and naturally occurring human teratocarcinomas, where multiple foci co-expressing the proliferation marker Ki-67 and the stem cell marker Oct3/4 were detected (Gidekel et al., 2003). These observations suggest that hESCs fully differentiate when transplanted into immunodeficient mice, posing a very low risk of malignant growth. However, genomic aberrations acquired by hPSCs during culture, some of which are also present in cancer cells, might increase the risk of uncontrolled proliferation and malignant transformation of differentiated hPSC derivatives, necessitating continuous and careful monitoring of genetic integrity of hPSC-based therapeutic preparations (Andrews et al., 2022; Keller and Spits, 2021).

Given that the direct injection of hPSCs into recipient animals results in tumorigenicity (Bedel et al., 2017), it is essential to differentiate PSCs before transplantation and then transplant only a purified population of differentiated derivatives. Several studies have indicated a considerable risk of teratoma formation following the injection of PSC-derived cells. For example, teratocarcinomas were observed after transplantation of mouse ESC-derived neural progenitor cells into the adult mouse hippocampus, even after using fluorescence-activated cell sorting (FACS) to remove residual PSCs (Germain et al., 2012). Cui and colleagues showed that ocular tumors could form in mice receiving retinal progenitor cells derived from mouse ESCs for retinal degeneration treatment (Cui et al., 2013). As mentioned earlier, a rapidly growing and chemotherapy-resistant immature teratoma was recently reported in a patient following injection of autologous iPSC-derived pancreatic beta cells into the deltoid muscle. This clearly demonstrates that the risk of tumorigenicity is not an imaginary threat and could occur in any patient receiving iPSC-derived cells if these therapeutic products are not rigorously quality tested (Han et al., 2022). However, in studies using hPSC derivatives, teratomas have mostly been observed when pure hPSC populations were intentionally injected into immunodeficient mice, either locally or systemically, and rarely occur when differentiated cells derived from hPSCs are administered for therapeutic purposes. For instance, while teratomas developed in 11 out of 15 immunodeficient mice (73%) after subcutaneous administration of 10,000 hESCs mixed with 5 × 10^5^ feeder cells and Matrigel, only 1 of 4 mice (25%) developed tumors after the injection of 5 × 10^5^ neural progenitors contaminated with 16,000 hESCs in a mixture of 5 × 10^5^ feeder cells and Matrigel (Gropp et al., 2012). Lower teratoma formation efficiency (2 out of 5 mice, 40%) was also observed after transplantation of 5 × 10^5^ hESC-derived retinal pigment epithelial cells spiked with 10,000 undifferentiated hESCs and administered together with 5 × 10^5^ feeder cells (Gropp et al., 2012). This study suggests that differentiated hPSC derivatives may reduce the detection sensitivity of teratoma formation, which should be considered during preclinical quality control of hPSC derivatives. Furthermore, neural overgrowths—but not teratomas—were reported in different animal models of Parkinson’s disease after injection of hESC-derived dopaminergic (DA) neurons into striatum (Kriks et al., 2011; Roy et al., 2006). These tumors consisted of proliferating primitive neuroectodermal cells within the graft (Doi et al., 2012; Kriks et al., 2011) and were observed only after transplantation of immature or rosette-derived DA neurons but not after the administration of DA neurons that had undergone prolonged *in vitro* maturation prior to transplantation (Doi et al., 2012) or those generated using an improved floor-plate-based differentiation strategy (Kriks et al., 2011). Using the optimized differentiation protocol in the Good Manufacturing Practice-compliant manufacturing process of hPSC-derived DA neurons, it was possible to eliminate the tumorigenic risk in a recent safety study in a rat model of Parkinson’s disease as assessed 39 weeks after cell transplantation (Kirkeby et al., 2023). This study also showed that the transplanted cells mediated full functional recovery in diseased animals, paving the way for the use of this cell product in patients with moderate Parkinson’s disease that was initiated in 2022. Table 1 lists the studies in which the formation of unwanted tumor-like structures was observed upon the transplantation of PSC-differentiated cells. Tumorigenicity associated with PSC-based cell therapy, although rare, is a significant phenomenon (Han et al., 2022). Therefore, cell manufacturers and clinicians involved in hPSC-based cell therapies must seriously consider this risk. By implementing strategies to effectively remove residual undifferentiated hPSCs, this risk can be minimized, thereby maximizing the safety of cell therapy.

**Table 1:** Studies reporting the formation of tumors or tumor-like structures upon transplantation of human and murine PSC-derived cells

Cell sources	Transplanted cells	Animal models	Site of transplantation	Number of injected cells	Tumor type	Reference
Human ESCs	dopaminergic neurons	rat	brain	500,000	teratomas	Roy et al., 2006
Human ESCs	dopaminergic neurons	mouse, rat, and monkey	brain	150,000 (mice), 250,000 (rats), 7,500,000 (monkeys)	neural overgrowths	Kriks et al., 2011
Mouse ESCs	neural progenitor cells	mouse	brain (hippocampus)	50,000	teratocarcinomas	Germain et al., 2012
Human ESCs	dopaminergic neurons	monkey	brain	400,000	non-teratomatous brain tumors	Doi et al., 2012
Mouse ESCs	retinal progenitor cells	mouse	subretinal space	80,000	neural and ocular tumors	Cui et al., 2013
Human iPSCs	neural progenitor cells	mouse	lesion epicenter	500,000	Nestin^+^ neural tumors	Nori et al., 2015
Human iPSCs	pancreatic progenitor cells	mouse	kidney	1,000,000	teratocarcinoma-like tumors	El Khatib et al., 2016
Human iPSCs	neural progenitor cells	mouse	lesion epicenter	500,000	ND	Kojima et al., 2019
Human iPSCs	pancreatic beta cells	human	deltoid muscle	not reported	immature teratomas	Han et al., 2022

## Potential mechanisms of PSC tumorigenicity

The mechanisms responsible for the tumorigenicity of PSCs have not been fully elucidated. PSCs may form teratomas as a natural consequence of their intrinsic ability to actively proliferate and differentiate into various tissues during embryonic development. However, PSCs also possess signaling characteristics that could facilitate, or even induce, the formation of teratomas or teratocarcinoma. For example, in mice, the ERAS gene is highly expressed in ESCs, where it constitutively activates phosphatidylinositol 3-kinase signaling. Interestingly, inactivation of ERAS does not alter the self-renewal or pluripotency of ESCs but significantly reduces their capacity to form teratomas or teratocarcinomas (Takahashi et al., 2003). Mouse PSCs are also unique in their regulation of the cell cycle. Their mitotic cycle is characterized by the continuous high expression of cyclin E:Cdk2 and cyclin A:Cdk2 complexes, which leads to the permanent hyperphosphorylation of retinoblastoma (Rb) protein, independent of cyclin D:Cdk4 and cyclin D:Cdk6 complexes (Sage et al., 2000; Savatier et al., 1994, 1996; Stead et al., 2002; White et al., 2005). Furthermore, mouse ESCs lack a DNA damage checkpoint in the G1 phase, unlike somatic cells (Aladjem et al., 1998; Hong and Stambrook, 2004). This suggests that the mechanisms regulating the G1 to S phase transition in response to growth signals are impaired or absent in mouse ESCs, which might greatly facilitate teratocarcinoma formation when these cells are introduced in an ectopic site. The cell cycle of hPSCs exhibits slightly different characteristics. These cells do not express ERAS and remain dependent on the Rb pathway for controlling the G1 to S phase transition (Conklin et al., 2012; Kareta et al., 2015). However, like their murine counterparts, hPSCs lack the DNA damage checkpoint in the G1 phase (Momcilovic et al., 2009, 2010). This indicates that the cell cycle of hPSCs, unlike that of mouse ESCs, retains at least some of the regulatory mechanisms typically observed in somatic cells. This difference in cell cycle regulation between mouse and hPSCs raises the question of whether it underlies the observation that hPSCs form teratomas but not teratocarcinomas.

It should be noted that conventional human and mouse PSCs do not self-renew in the same pluripotency state. Mouse ESCs self-renew in the so-called naive state of pluripotency, an immature state corresponding to the early epiblast of the blastocyst. In contrast, conventional hPSCs, whose cell cycles have been investigated in the aforementioned studies, self-renew in the primed state of pluripotency, which is a less immature state observed in the late epiblast of the gastrulating embryo (Endoh and Niwa, 2022; Nichols and Smith, 2009; Taei et al., 2020). Therefore, it would be highly informative to investigate the regulation of the cell cycle in newly described naive hPSCs. Specifically, it would be interesting to determine whether these naive (or naive-like) hPSCs can form transplantable teratocarcinomas, similar to their murine counterparts, when injected into SCID or NUDE mice.

PSC tumorigenicity may arise from genetic abnormalities in PSCs caused by *in vitro* cultivation and experimental manipulation. These changes can alter the properties of PSCs and potentially increase their tumorigenic risk. Therefore, it is crucial to develop culture conditions that minimize genetic instability during PSC cultivation and differentiation *in vitro*. Studies suggest that PSC teratoma formation may result from specific genetic and epigenetic instabilities (Andrews et al., 2022). Regarding genetic instability, the genes *SOX2*, *OCT3/4*, *c-MYC*, and *KLF4*—commonly used for reprogramming somatic cells to iPSCs—are often expressed at higher levels in various cancer cells and are involved in the development and progression of several cancers (Lezmi and Benvenisty, 2022). For example, Gidekel and colleagues demonstrated that the tumorigenic potential of hPSCs is influenced by OCT3/4 in a dose-dependent manner, showing that non-tumorigenic cells can be transformed into tumorigenic ones through OCT3/4 overexpression (Gidekel et al., 2003). The oncogenic properties of Oct3/4 were also investigated by ectopically expressing it in somatic tissues of adult mice (Hochedlinger et al., 2005). This study revealed that Oct3/4 activation induces dysplasia in various epithelial tissues by inhibiting the differentiation of progenitor cells. Additionally, OCT3/4 has been found to be upregulated in several human cancers and is associated with poor prognosis in patients with these cancers (Feng et al., 2019; Rasti et al., 2018; Xie et al., 2022).

In an effort to determine how the expression of core pluripotency-associated transcription factors—OCT4, NANOG, SOX2, KLF4, and c-MYC—correlates with tumor aggressiveness, Porath and colleagues found that the expression of ESC transcription regulators in tumors was negatively correlated with the degree of tumor differentiation (Ben-Porath et al., 2008). Another study investigated the effect of L-Myc on promoting iPSC generation using retroviral vectors. The authors reported that replacing c-MYC with L-MYC, another member of the MYC family with lower tumorigenic potential, improved the efficiency of hiPSC formation, and no tumorigenesis was observed in chimeric mice generated with L-MYC mouse iPSCs (Nakagawa et al., 2010). This study also suggests that c-MYC is not the only reprogramming factor responsible for tumor development, as iPSC-derived tumors have been reported even in the absence of c-MYC. On the other hand, Miura et al. reported that the presence or absence of c-MYC in mouse iPSCs did not correlate with tumor size in NOD/SCID mice (Miura et al., 2009). In addition to c-MYC, other pluripotency factors such as OCT4, SOX2, NANOG, and KLF4 are known to act as potent cancer drivers. For example, CD24, which drives NANOG expression, has been identified as a liver cancer-causing gene with key features such as tumor initiation, self-renewal, chemoresistance, and differentiation (Lee et al., 2013b). Furthermore, SOX2 has been reported to play a central role in maintaining the oncogenic behavior of cancer cells in squamous cell carcinomas of the lung and esophagus, where both squamous differentiation and pluripotency gene markers are expressed by SOX2-driven tumors (Bass et al., 2009). These studies highlight that key transcriptional regulatory factors of PSCs are significant drivers of major cancer phenotypes.

iPSC tumorigenicity also appears to originate from epigenomic instability, primarily due to DNA methylation (Iida et al., 2017). Naive and primed PSCs are epigenetically characterized by a global demethylation pattern (Geng et al., 2019). However, hypermethylation of tumor suppressor genes and hypomethylation of oncogenes in hPSCs can contribute to tumorigenicity (Zhang et al., 2012). Tumors may also arise from differentiated cells due to accumulation of genomic aberrations and mutations acquired during *in vitro* passaging and adaptation in culture. The mutations found are among the most common mutations in human cancers and may increase the risk for tumor formation (Gore et al., 2011; Merkle et al., 2017). Additionally, the use of integrative vectors or viruses, such as lentiviruses or retroviruses, can lead to genomic instability (Kang et al., 2015). This instability may result from the disruption of tumor suppressor genes, integration-induced hyperactivation of proto-oncogenes, or alterations in signaling pathways following vector or viral integration into the genome, all of which can potentially lead to tumor formation.

## Approaches to eliminating undifferentiated PSCs

**Figure 2:**
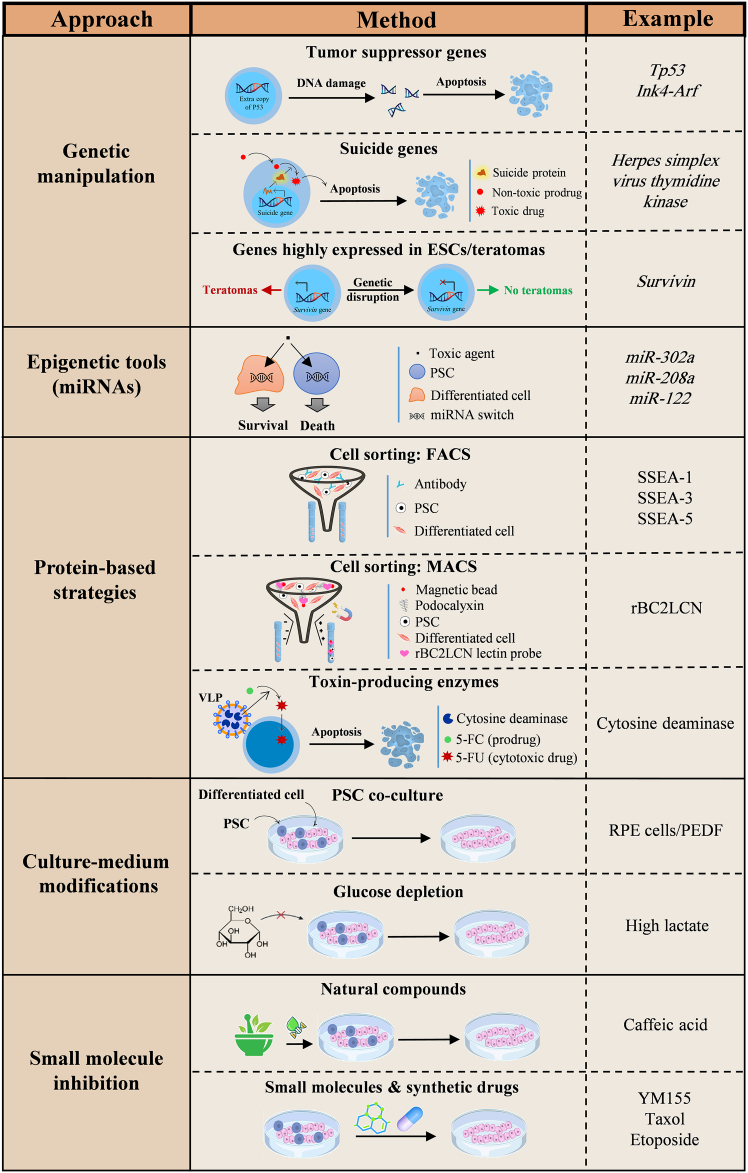
Strategies for the elimination of undifferentiated PSCs The strategies that were developed for elimination of undifferentiated PSCs from differentiated PSC derivatives include insertional (e.g., overexpressed transgenes) and non-insertional (e.g., microRNA switches) genetic approaches, employing protein-based techniques, such as antibodies or toxins, modification of the culture media during the differentiation and purification stages in the production of hPSC-based therapeutics, and treatment with natural or synthetic chemical compounds. 5-FU, 5-fluorouracil; 5-FC, 5-fluorocytosine.

Several strategies have been developed to eliminate tumorigenic cells from differentiated PSC derivatives (Figure 2), which can be grouped into the following categories: insertional genetic manipulation, insertion-free genetic approaches, antibody- or toxin-based depletion, selective cell culture conditioning, and treatment with PSC-toxic small-molecule compounds.

## Insertional genetic approaches

Early studies aimed at inhibiting PSC growth and tumorigenesis often focused on the overexpression of exogenous suicide genes in PSCs. A suicide gene is one that triggers cell death through multiple pathways including apoptosis, upon activation. This strategy involves delivering a transgene encoding a specific enzyme into the cells, followed by treating these cells with a prodrug, which the enzyme converts into a cytotoxic drug. The most prominent examples of suicide gene systems include the herpes simplex virus thymidine kinase (HSV-*tk*) in combination with the prodrug ganciclovir, and the inducible caspase-9 (iCASP9) gene combined with a small molecule called a chemical inducer of dimerization (CID), which is required for caspase-9 dimerization to activate the downstream caspase-3/7 and induce apoptosis (Itakura et al., 2017; Liu et al., 2022; Shi et al., 2020; Wunderlich et al., 2022). The HSV-*tk*/GCV system has been extensively used in cancer cell ablation studies for over 30 years (Fillat et al., 2003) and has also been safely applied in the clinical studies within the context of adoptive T cell therapy (Zhan et al., 2013). In 2003, Schuldiner and colleagues were the first to genetically modify hESCs to constitutively express an HSV-*tk* under the control of the housekeeping phosphoglycerate kinase promoter. HSV-*tk* phosphorylates and activates ganciclovir, enabling its incorporation into the replicating DNA of hPSCs during S phase, leading to G2/M arrest and apoptosis. Even under prolonged selection with ganciclovir, the reversion rate of engineered cells remained low (Schuldiner et al., 2003). As an alternative, Zhao et al. developed an episomal suicide construct for the expression of an HSV-*tk* suicide gene under the control of the *OCT4* promoter, which is specifically active only in hPSCs. Human pluripotent embryonic carcinoma cells (i.e., NCCIT cell line) were exposed to retinoic acid to induce differentiation and then treated with or without ganciclovir. Subsequently, 3 × 10^6^ differentiated cells from both treatment groups were injected into SCID mice. Interestingly, while 6 out of 9 mice injected with untreated cells developed tumors after 3 months, only 1 out of 12 mice injected with ganciclovir-treated cells did (Zhao et al., 2012). However, the specific and complete elimination of tumorigenic hPSCs via single-gene suicide constructs may not always be achieved, and the application of multiple suicide genes might provide superior PSC removal.

Recently, a two-plasmid approach was introduced to generate genetically modified cells using tumorigenic cell-targeting lentiviral vectors. These vectors contain various promoters, such as the survivin-encoding gene (*BIRC5*) promoter and the cytomegalovirus enhancer/β-actin (*CA*) hybrid promoter, upstream of a fluorescent protein and the HSV-*tk* suicide gene (Ide et al., 2018). Using this two-plasmid system, ganciclovir induced significant cytotoxicity in HSV-*tk*-transduced hPSCs, and the authors achieved high specificity for undifferentiated hPSCs using the *survivin* promoter. Additionally, ganciclovir prevented the genetically modified hPSCs from forming teratomas when injected into immunodeficient mice (Ide et al., 2018). Notably, survivin is abundantly expressed in hESCs and teratomas, and suppressing survivin using small-molecule chemicals or genetic disruption can also be used to inhibit hPSC growth and teratoma formation (Blum et al., 2009).

The iCASP9 system, which merges human caspase-9 with a modified human FK506-binding protein, becomes active upon exposure to a CID such as AP1903. Once activated, iCASP9 induces rapid apoptosis specifically in cells expressing this construct, effectively serving as a “safety switch” for selectively eliminating cells when necessary (Di Stasi et al., 2011; Straathof et al., 2005). Shi et al. successfully installed the iCASP9 suicide gene system into the AAVS1 genome safe-harbor locus of hiPSCs, leading to more efficient iCASP9 expression driven by the *CAG* promoter. Upon AP1903 treatment, the death of hiPSCs was induced, halting cell growth within 10 days *in vitro*. Importantly, the engineered hiPSCs retained their differentiation potential into various cell types, preserving their therapeutic capabilities. Teratoma formation was ablated in NSG mice treated with AP1903 (Shi et al., 2020). Another study focused on the AAVS1 safe-harbor locus in hiPSCs for monoallelic and biallelic integration of iCASP9, resulting in apoptotic death of hiPSCs *in vitro* after CID administration. *In vivo* experiments with NOD/SCID mice demonstrated that CID administration eliminated cystic teratomas in three out of five mice injected with transgenic monoallelic iCASP9 hiPSCs. However, individual animals subsequently formed tumors from monoallelic iCASP9 hiPSCs (Wunderlich et al., 2022). In 2017, Itakura et al. transduced iCASP9 under the elongation factor-1α (EF-1α) or ubiquitin C (UbC) promoter into two hiPSC lines using a lentiviral vector. The system’s efficacy was evaluated *in vitro*, demonstrating that 95% of the iCASP9-hiPSC and hiPSC-derived neural stem/progenitor cells (hiPSC-NS/PCs) underwent apoptosis after culturing with CID (AP20817). Additionally, teratoma ablation was reported in NOD/SCID mice following the transplantation of both hiPSC-NS/PCs cell lines into injured spinal cords (Itakura et al., 2017). However, the system’s lack of specificity through the use of ubiquitously active promoters driving the transgene expression, affecting both undifferentiated and differentiated cells, limits its clinical utility. Future improvements should focus on lineage-specific targeting to preserve therapeutic progenitors while eliminating tumorigenic hPSCs. In a recent study, Liu et al. inserted the *iCASP9* gene downstream of the endogenous *OCT4* locus in both human and mouse PSCs. Activation of iCASP9 by AP1903 led to apoptosis of undifferentiated human and mouse PSCs *in vitro*. Furthermore, mouse and human OCT4-iCasp9 PSCs were selectively eradicated by AP1903 within 2 weeks after PSC injection in immunodeficient mice. However, if the interval exceeded 2 weeks, teratomas formed from differentiated somatic cells derived from the PSCs (Liu et al., 2022).

Another approach involves the use of a photosensitizer suicide gene system, which allows controlled cell death upon light activation. Cho et al. used KillerRed (KR), an artificial photosensitizer protein activated by visible light (Cho et al., 2015). A single exposure to visible light selectively eliminated undifferentiated mouse KR-overexpressing PSCs while preserving the functionality of endothelial cells derived from KR-PSCs *in vitro*. Furthermore, visible light exposure inhibited teratoma formation in nude mice injected subcutaneously or intratesticularly with KR-PSCs. Interestingly, blood perfusion increased in ischemic hindlimbs injected with endothelial cells derived from KR-ESCs, whereas the muscles of PBS-injected mice displayed massive degeneration, abnormal structures, and fibrosis due to femoral artery occlusion. These observations confirm the effective regenerative ability of these cells *in vivo* (Cho et al., 2015).

In addition to suicide genes, the doxycycline-inducible overexpression of the plasma membrane Na^+^/H^+^ exchanger 1 (NHE1) has been reported to induce necrotic death of hiPSCs but not mesendoderm-like cells via activation of Rho-associated protein kinase (ROCK) (Wakabayashi et al., 2019). Interestingly, selective killing of undifferentiated hiPSCs was also achieved by treatment with the antibiotic monensin, which behaves like a Na^+^/H^+^ antiporter in the plasma membrane. However, the mechanism of action does not appear to rely solely on the enhancement of ROCK activity, as monensin-induced hiPSC death was not significantly blocked by the ROCK inhibitor Y27632 (Wakabayashi et al., 2019). While this study showed that early mesendoderm-like progenitor cells are resistant to NHE1 toxicity, it is unclear whether this is true for other, more differentiated therapeutically relevant cells, which could limit their clinical use.

Finally, another study took advantage of the tumor-suppressing roles of the *Tp53* gene and the *Ink4/Arf* locus, showing that mouse embryonic fibroblast (MEF)-derived iPSCs with an extra copy of these genes exhibited a lower tendency to form tumors compared to normal iPSCs (Menendez et al., 2012). However, this study did not demonstrate whether increased dosage of p53 or inhibitor of N-linked K-dependent 4-kinase 4a (Ink4a)/alternative reading frame (ARF) would similarly reduce the tumorigenic potential of hiPSCs. While this model is suitable for basic studies, its application in reducing the tumorigenic propensity of cells needed for clinical use is limited, as it would require genetic manipulation.

While PSC removal strategies based on genetic manipulation have shown promise, the long-term consequences of such genetic alterations remain unknown. Although genome-editing technologies now enable the precise insertion of transgenes into specific genomic regions for clinical use (Liu et al., 2022; Wunderlich et al., 2022), such alterations might disrupt critical genes or important genomic regions involved in tumorigenesis, necessitating long-term monitoring of patients for potential genetic risks. Additionally, the constitutive expression of viral or foreign non-human suicide genes could lead to immunogenicity, increasing the risk of immune rejection of cells expressing them. Furthermore, suicide transgenes could acquire inactivating mutations over time, as seen with the HSV-*tk* gene, rendering hiPSCs resistant to ganciclovir and increasing the risk of teratoma formation (Kotini et al., 2016). These concerns highlight the need for safer strategies to eliminate undifferentiated PSCs.

## Insertion-free genetic approaches

This approach involves transfecting synthetic, *in vitro*-transcribed mRNA encoding a protein—such as a fluorescent protein, an antibiotic resistance enzyme, an apoptosis inducer, or a cell surface receptor—into the target cell population (Miki et al., 2015; Tsujisaka et al., 2022). The translation of the transfected mRNA is controlled by a microRNA (miRNA) switch, which is positioned in the 5′ untranslated region (UTR) of the synthetic mRNA. This switch comprises a sequence complementary to a specific endogenous miRNA. In cells expressing the corresponding endogenous miRNA, the miRNA binds to the complementary sequence in the synthetic mRNA, leading to translational repression of the target mRNA. Conversely, in cells lacking the corresponding miRNA, the transfected synthetic mRNA is translated, with the extent of translation depending on the level of miRNA expression.

miRNAs are small non-coding RNAs that regulate gene expression at the post-transcriptional level, influencing various cellular processes, including proliferation, apoptosis, migration, self-renewal, and differentiation (Divisato et al., 2020; Moradi et al., 2014; Saliminejad et al., 2019). In PSC biology, some miRNAs support pluripotency, while others promote differentiation (Divisato et al., 2021; Kulcenty et al., 2019; Moradi et al., 2017, 2023). The miRNA switch system has been used to identify, purify, and/or eliminate specific cell populations based on endogenous miRNA activity. For example, a miRNA switch sensing the pluripotency-associated miR-302a-5p (miR-302a) was utilized to specifically eliminate undifferentiated hPSCs (Parr et al., 2016). In this case, the miR-302a target sequence was placed in the 5′ UTR of synthetic mRNA encoding puromycin N-acetyl-transferase (PAC), which confers resistance to the antibiotic puromycin. In hPSCs transfected with this miRNA switch construct (∼95% transfection efficiency), endogenous miR-302a represses PAC expression, allowing for a specific removal of undifferentiated hPSCs upon puromycin treatment. Interestingly, a miR-302a switch driving the expression of a fluorescent protein, rather than PAC, was also able to identify and purify partially differentiated cells. These cells still expressed miR-302a, although to a lesser extent than undifferentiated hPSCs. Notably, isolating hiPSCs from a mixed culture using the miR-302a switch was more sensitive than FACS based on the TRA-1-80 cell surface marker, as TRA-1-80 expression diminishes more rapidly upon differentiation compared to miR-302a. Moreover, other hPSC-enriched miRNAs, such as miR-302c-3p, miR-302d-5p, miR-518f-5pp, and miR-519a-3p, have been identified as markers for detecting and distinguishing hiPSCs within differentiated populations of various immune cell types. They could be used to enhance the safety of cell therapy using hiPSC-derived NK cells (Chung et al., 2022). The miRNA switch system also holds great potential for producing pure differentiated cells for cell therapy. For instance, Miki et al. used miR-1-, miR-208a-, and miR-499a-5p switches to efficiently purify hPSC-derived cardiomyocytes and utilized switches encoding the apoptosis inducer Bim to enrich cardiomyocytes without the need of cell sorting (Miki et al., 2015). In addition, the same research group employed miR-126, miR-122-5p, and miR-375 switches to purify hPSC-derived endothelial cells, hepatocytes, and insulin-producing cells, respectively.

The advantage of the miRNA switch strategy lies in its safety, as it does not require stable genomic integration of the transfected genetic material, and mRNA transfection is minimally toxic to cells. Moreover, the mRNA can be rapidly developed and is more cost-effective compared to several other technologies. However, miRNA switches are only transiently expressed, allowing for cell detection and isolation within a narrow time frame. This transience may be a disadvantage, potentially necessitating multiple transfections to achieve the desired purification or elimination efficiency. Furthermore, transfection efficiency might be significantly reduced in densely growing cell cultures typical of many hPSC differentiation procedures or in large-volume bioreactors where cells grow as three-dimensional aggregates. Under these conditions, the ability to fully eliminate undesired and potentially hazardous cell populations could be compromised. Moreover, there is a risk that the reporter mRNA could be reverse transcribed into cDNA and subsequently integrated into the genome, compromising genomic integrity. This risk arises not only from endogenous LINE-1 (L1) retrotransposons—which encode a reverse transcriptase (L1-ORF2p) capable of converting exogenous RNA into cDNA (Sciamanna et al., 2016)—but also from DNA polymerase theta (Polθ). Recent studies demonstrate that Polθ functions as an efficient reverse transcriptase, exhibiting higher velocity and fidelity on RNA templates than on DNA, and even promotes RNA-templated DNA repair in mammalian cells (Chandramouly et al., 2021). Finally, while the miR-302a switch was reported to have a 97% positive response for eliminating hPSCs, the remaining 3% could still pose a risk to cell therapy, since as few as 20–100 undifferentiated PSCs could cause teratoma formation (Lawrenz et al., 2004; Parr et al., 2016). Therefore, further research is required to effectively harness miRNAs for efficient PSC elimination.

## PSC depletion strategies based on antibodies

Monoclonal antibodies (mAbs) targeting specific PSC surface markers can distinguish undifferentiated PSCs from other cells, making them valuable tools for eliminating undifferentiated cells from differentiated cell populations. In 2011, it was reported that an stage-specific embryonic antigen-5 (SSEA-5) mAb could effectively remove undifferentiated hPSCs via FACS, thereby reducing teratoma formation (Tang et al., 2011). However, SSEA-5 and other pluripotency-associated markers are not exclusively expressed in hPSCs. In an effort to address this limitation, Choo and colleagues aimed to produce hPSC-specific antibodies. They immunized BALB/c mice with live hESCs and, from a group of 10 mAbs, identified mAb 84, which specifically recognized and was cytotoxic to hESCs but not to differentiated cells. This mAb was found to target the podocalyxin-like protein-1 (PODXL) on the cell surface (Choo et al., 2008). Injection of hESCs treated with this antibody into the hind leg muscle of male SCID mice completely prevented teratoma formation, as determined by visual monitoring of the animals over 18–24 weeks. Subsequent studies by the same group revealed that this antibody exerts its cytotoxic effect within minutes via oncosis, a process characterized by membrane damage, pore formation, and cell swelling (Tan et al., 2009). The toxicity of PODXL-specific antibodies against hPSCs was further confirmed in a later study, which highlighted that targeting PODXL offers an advantage over targeting TRA-1-60 or TRA-1-81 cell surface epitopes. While TRA-1-60 and TRA-1-81 are lost early in differentiation, PODXL remains expressed in a subpopulation of potentially tumorigenic cells, even after prolonged differentiation. This suggests that antibodies against PODXL could more efficiently remove undifferentiated hPSCs and significantly reduce the risk of tumor formation (Kang et al., 2016). In addition to PODXL-antibodies, the TAG-A1 mAb, which recognizes an O-linked glycan epitope on multiple hESC and hiPSC cell surface antigens, also kills undifferentiated hPSCs via oncosis (Zheng et al., 2017). This antibody increases the production of reactive oxygen species (ROS) in hPSCs by activating death signaling through dimerization of antigen receptors, leading to microvilli degradation and homotypic cell adhesion.

Another strategy for elimination of hPSCs involves mAbs against desmoglein 2 (DSG2) that is expressed on the surface of hPSCs (Park et al., 2018, 2020). In this approach, the DSG2-specific mAb K6-1 is covalently bound to the chemotherapeutic agent doxorubicin, forming an antibody-drug conjugate. Upon incubation with hESCs, the conjugate is internalized by endocytosis and induces apoptosis without major side effects on human fibroblasts. Pre-treatment of hESCs with the antibody-doxorubicin conjugate suppressed teratoma formation after injection into the testes of immunodeficient NSG mice. However, the effects on other types of differentiated cells were not investigated, weakening the claim of selectivity. Moreover, the conjugate’s efficacy in a more clinically relevant scenario, such as in transplants of differentiated cells mixed with varying amounts of undifferentiated hESCs, remains unclear. Finally, DSG2 is a known component of desmosomes that form cell-cell contacts in various differentiated cell types, such as epithelial cells and cardiomyocytes. Therefore, targeted elimination of hPSCs by this antigen may only be suitable for hPSC derivatives that do not express DSG2.

The eradication of tumorigenic hPSCs was also achieved by targeting CD30 with the Food and Drug Administration (FDA)- and European Medicines Agency (EMA)-approved antibody-drug conjugate called brentuximab vedotin, commonly used to treat Hodgkin lymphoma and other lymphomas (Sougawa et al., 2018). This antibody is covalently linked via cathepsin-cleavable linkers to the antimitotic agent monomethyl auristatin E (Francisco et al., 2003). Upon binding to CD30-expressing hPSCs, the conjugate is internalized by endocytosis and cleaved within lysosomes, releasing the drug that kills the cells. Sougawa and coworkers demonstrated that brentuximab vedotin is toxic to hiPSCs and that transplantation of hiPSC-derived cardiomyocytes treated with this drug does not lead to tumor formation, suggesting its potential for increasing the safety of hPSC-based therapies. These findings were corroborated by Pellegrini et al. who showed that teratoma development after transplantation of iPSC-derived β cells can be prevented by pre-treatment with brentuximab vedotin (Pellegrini et al., 2022). Given that CD30 is also expressed on various cancer cells, this drug could potentially treat tumors other than teratomas resulting from malignant transformation of transplanted hiPSC derivatives.

Ben-David and coworkers leveraged the hPSC-specific expression of the tight-junction protein claudin-6 for hPSC elimination using three different approaches (Ben-David et al., 2013b): (1) labeling hPSCs with claudin-6 mAb followed by FACS, (2) killing hPSCs with claudin-6 mAb conjugates and secondary saporin toxin-conjugated antibodies, and (3) treating cells with the pore-forming *Clostridium perfringens* enterotoxin (CPE) that specifically binds claudin-6. The latter method proved extremely efficient, inducing cell death in virtually all undifferentiated hPSC in culture within 1 hour, while fibroblasts, cardiomyocytes, and neuronal cells remained unaffected. This effect was confirmed *in vivo*, as no teratomas formed in immunodeficient NOD-SCID-IL2Rγ^−/−^ mice after subcutaneous injection of one million CPE-treated differentiated cells. Of note, since claudin-6 is also expressed in some differentiated cells, the utility of this marker would be context dependent. Another obstacle to the more widespread use of *Clostridium* enterotoxin is the safety issue due to the risk of systemic toxicity if the toxin is ingested, inhaled, absorbed through damaged skin, or transplanted with therapeutic cells treated with it.

mAbs against specific cell surface markers have also been utilized to eliminate tumorigenic cells from differentiated PSC derivatives through magnetic-activated cell sorting (MACS) after conjugation to magnetic beads. For example, Kahan and coworkers developed a three-stage method using three different mAbs to remove unwanted cell types from endodermally differentiated murine ESC cultures, followed by positive selection of desired cells (Kahan et al., 2011). In the first step, murine undifferentiated ESCs were removed with SSEA1 mAbs, followed by sorting out extraembryonic endoderm cells from the SSEA1-negative population using SSEA3 mAbs. In the final positive selection step, definitive endoderm cells were enriched using mAbs against epithelial cell adhesion molecule (EpCAM). When transplanted into immunocompromised mice, purified murine EpCAM^+^ SSEA1^−^ SSEA3^−^ cells did not produce teratomas, even after 23 weeks of follow-up. This suggests that targeting multiple undifferentiated and differentiated cell markers may better minimize hPSC tumor formation risk, especially compared to methods relying on genetic manipulation. Although this three-step method for the purification of definitive endoderm cells was also demonstrated with human ESCs, experimental evidence showing that purified human cells do not form teratomas is lacking. In addition, cell sorting based on surface markers may pose challenges for clinical applications, as it requires enzymatic dissociation, which could affect cell yield, viability, and functionality. Furthermore, this approach may not be applicable to cell aggregates that cannot be dissociated into single cells prior to transplantation. Methods based on single-cell sorting may also fail to completely eliminate the risk of teratoma formation, as the efficiency of removing residual hPSC is likely to be compromised when large quantities of cells are required for therapeutic purposes. Sorting cells with FACS or MACS might also introduce microbial contamination, compromising the safety of cell therapy.

Using antibodies is appealing due to their high selectivity. However, antibodies are expensive, may cause immune responses, and the use of FACS or MACS can be challenging and prone to inefficiencies. This makes their application costly, and it is difficult to ensure that all undifferentiated hPSCs will be removed, especially in situations where hundreds of millions of differentiated cells are needed for therapeutic applications. In addition, antibodies could elicit an immune response if they remain bound to transplanted cells, potentially jeopardizing their therapeutic efficacy. It should be noted that most hPSC markers mentioned earlier are also expressed in some differentiated cell types. Therefore, the selection of a specific marker depends on ensuring that it is not expressed in the cells of the lineage of interest for the given application.

## PSC depletion strategies based on lectins and toxins

In addition to antibodies, other proteins have been explored for removing tumorigenic PSCs. Lectins, which bind specific carbohydrate groups of glycoproteins or glycolipids, can label cells for various purposes. The lectin probe called rBC2LCN (recombinant N-terminal domain of BC2L-C lectin derived from *Burkholderia cenocepacia*) specifically recognizes the transmembrane glycoprotein podocalyxin on undifferentiated hiPSCs but does not bind differentiated cells. Tateno and coworkers developed a soluble recombinant form of this protein fused to different catalytic domains of *Pseudomonas aeruginosa* exotoxin A. This fusion protein is internalized by and efficiently kills hESCs and hiPSCs but not human fibroblasts, MSCs, retinoic acid-treated hiPSCs, or hiPSC-derived hepatocytes (Tateno et al., 2015, 2017). Although the ability of this strategy to prevent teratoma formation *in vivo* was not tested, a follow-up study by the same group showed that the rBC2LCN probe, when conjugated to magnetic beads, could eliminate undifferentiated hiPSCs from a mixture of adult human dermal fibroblasts and hiPSCs using MACS (Haramoto et al., 2020). The residual hiPSC contamination was assessed by droplet digital reverse-transcription PCR, showing that approximately 99.8%–100% of iPSCs were eliminated. Importantly, the purified differentiated cells did not form tumors upon injection into the testes of immunodeficient mice, demonstrating the high potential of this strategy for reducing the tumorigenic risk of hPSC derivatives. However, this method was only tested with a limited number of hiPSCs mixed with adult human fibroblasts, and its efficiency has not been validated in more complex cultures required for large-scale production of therapeutic cells. Additionally, concerns about the *in vivo* toxicity and immunogenicity of residual lectin-toxin fusion protein could further diminish the applicability of this approach for the preparation of therapeutic cells.

To enhance the efficiency and specificity of delivering suicide-inducing constructs to hPSCs, virus-like particles (VLPs) have recently been used as delivery vectors. VLPs are particles composed of self-assembling viral envelope proteins that mimic the structure of original viruses but cannot replicate because they lack a genome and replicases (Mohsen and Bachmann, 2022). They can be packaged with nucleic acids and proteins or engineered to carry specific epitopes on their surface. VLPs have mainly been used as highly efficient delivery vehicles for pharmaceutical products or as platforms for vaccine development. However, to deliver cytotoxic molecules to hPSCs, Rampoldi and coworkers engineered VLPs from Qβ bacteriophage capsids to contain cytosine deaminase in their core, which converts the prodrug 5-fluorocytosine into the cytotoxic drug 5-fluorouracil, and multiple *Staphylococcus aureus* protein A-derived Z-domains on their surface, which bind the Fc region of IgG antibodies (Rampoldi et al., 2018). To specifically target hPSCs, the cytosine deaminase-containing and Z-domain-displaying VLPs were labeled with antibodies against the hPSC-specific surface glycan SSEA-5 and added to co-cultures of hiPSCs and MEFs or hiPSC-derived cardiomyocytes. Treatment of these co-cultures with the prodrug 5-fluorocytosine led to a selective and complete elimination of undifferentiated hPSCs without adversely affecting the viability and functionality of terminally differentiated cells. The safe, selective, and scalable nature of this approach, which does not require genetic modification of the cells, makes it a promising strategy for eliminating the risk of tumorigenesis from various therapeutic hPSC-derived cell populations. Notably, some differentiated cells may express the aforementioned lectins. Therefore, as with antibodies, the selection of a specific lectin depends on ensuring that it is not expressed in the cells of the lineage of interest.

## Small molecules with big impacts

### Exploiting anti-apoptotic pathways

Using small molecules to eliminate PSCs from differentiated cells represents an effective strategy. Analysis of genes commonly expressed in teratomas and hESCs identified several potential targets for eliminating undifferentiated hESCs. Among these, *BIRC5* encoding for survivin emerged as the most promising candidate. Suppression of Survivin by combining the chemotherapeutic agent Taxol (paclitaxel) and the selective cyclin-dependent kinase inhibitor purvalanol A induced apoptotic death in hESCs (Blum et al., 2009). By comparing the expression profiles of pro- and anti-apoptotic genes in hESCs and their differentiated derivatives, Lee et al. found that the anti-apoptotic proteins survivin and BCL10 are highly expressed in hESCs. Inhibition of survivin by the flavonoid quercetin, found in many plants, or the structurally related small molecule YM155, suppressed hPSC survival and inhibited tumorigenesis, likely by inducing p53 accumulation in the mitochondria of hPSCs and activating the intrinsic apoptotic pathway (Lee et al., 2013c). Interestingly, YM155 has also been shown to enable efficient hepatic cell purification by selectively removing undifferentiated hPSCs during multi-step hepatocyte differentiation. YM155-purified hepatocytes retained their typical characteristics and did not form teratomas after transplantation into immunodeficient mice (Kang et al., 2017). Notably, a sub-population of high-passage hESCs was found to be resistant to YM155 due to upregulation of the *BCL2L1* gene and its anti-apoptotic protein product BCL-x. To overcome this resistance, Cho et al. used BH3 mimetics, such as ABT-737 and ABT-263, which induced apoptotic cell death in YM155-unresponsive hESCs by disrupting mitochondrial membrane potential (Cho et al., 2018). BH3 mimetics are small-molecule compounds that inhibit anti-apoptotic BCL-2 family of proteins, including BCL-xL, and have been used to induce apoptosis in various cancer cells (Delbridge and Strasser, 2015). However, since high-passage hESCs upregulating BCL2L1 could dominate the PSC culture, it is advisable to use a fresh batch of hPSCs for applications in regenerative medicine.

### Targeting lipid metabolism

Ceramides are waxy lipids composed of sphingosine joined to a fatty acid via an amide bond. They are involved in various physiological processes including cell differentiation, migration, and apoptosis. The cellular accumulation of ceramides has been observed upon exposure of cells to apoptotic agents, such as chemotherapeutic agents, tumor necrosis factor alpha, and ionizing radiation (Alphonse et al., 2013; Dbaibo et al., 2001; Martinez et al., 2012; Thomas et al., 2017), implicating this so-called “tumor suppressor lipid” as a mediator of apoptosis. Bieberich and colleagues found that prostate apoptosis response-4 (PAR-4), which is known to serve as a mediator of ceramide-induced apoptosis, colocalized with Oct4 in the cell nuclei during neural induction of mouse and human ESCs. They observed that treatment of embryoid body-derived cells with a ceramide analog called N-oleoyl serinol (S18) upon their transplantation into the mouse brain removed undifferentiated Oct4^+^/PAR-4^+^ cells through induction of ceramide-mediated apoptosis, enhanced the number of Nestin^+^ neural cells, and inhibited teratoma formation, as opposed to untreated transplants, which gave rise to several teratomas (Bieberich et al., 2004).

In 2013, Benvenisty and colleagues conducted high-throughput screening of 52,000 small molecules in hPSCs, identifying 15 compounds with hPSC-specific growth inhibitory effects. Of these, nine contained a phenylhydrazine structure, and a compound called PluriSIn #1 was found to be the most effective in inducing apoptosis, endoplasmic reticulum stress, and reducing protein synthesis in three different hESC lines and one hiPSC line but not in fibroblasts used to generate iPSCs, five different hPSC derivatives, or three cancer cell lines (Ben-David et al., 2013a). The molecular target of PluriSIn #1 was identified in an unrelated biochemical screen aimed at discovering new drugs for metabolic disorders, revealing that PluriSIn #1 exerts its selective cytotoxicity by inhibiting stearoyl-coA desaturase 1 (SCD1). This enzyme is an endoplasmic reticulum membrane protein that plays a key role in oleic acid production and lipid metabolism. The dependence of hPSCs on SCD1 activity for survival was further confirmed by treating hESCs with either structurally distinct SCD1 inhibitors or specific small interfering RNAs (siRNAs) against SCD1, both of which resulted in cell death. Interestingly, the cytotoxic activity of PluriSIn #1 against hPSCs decreased when cells were cultured in larger plates, at higher cell densities, or in the presence of feeder cells, suggesting that these factors must be carefully controlled to maintain the potency required for complete hPSC elimination. Nevertheless, *in vivo* experiments demonstrated that PluriSIn #1 effectively eliminates undifferentiated hPSCs in a mixture with differentiated cells and fully suppresses tumor formation when the remaining cells are injected subcutaneously into immunocompromised mice (Ben-David et al., 2013a). In a separate study, PluriSIn #1 was also found to have no negative effect on the growth of hPSC-derived cardiomyocytes in terms of *in vivo* engraftment and survival in the infarcted myocardium (Zhang et al., 2014), indicating that targeting lipid metabolism might be a viable option for restricting hPSC growth and ensuring the safety of hPSC-based therapies.

Additionally, it was reported that the diabetes mellitus drug metformin, which influences several aspects of fat metabolism (Rena et al., 2017), could reduce tumor formation by mouse PSCs in nude mice without harming their differentiation potential. This effect is mediated by AMP-activated protein kinase, for which metformin serves as an agonist (Vazquez-Martin et al., 2012). However, this study did not investigate hPSCs, which remains to be explored. Statins, well-known small-molecule drugs commonly prescribed to reduce low-density lipoprotein cholesterol in patients with hypercholesterolemia, have also shown promise. Specifically, atorvastatin, a member of the statin family, was reported to decrease the survival of hPSCs but not hPSC-derived cardiomyocytes, resulting in the inhibition of teratoma formation. Atorvastatin induced hPSC death through the hypoxia-inducible factor-1α-peroxisome proliferator-activated receptor-ɣ (HIF1α-PPARɣ) axis, which plays a crucial role in hPSC survival (Nakashima et al., 2018). These findings highlight that lipid metabolism can be exploited to enhance the safety of hPSC-based cell therapies.

### Targeting DNA

In 2021, Toru Kondo reported that the enzyme dihydroorotate dehydrogenase (DHODH), which plays a key role in *de novo* pyrimidine biosynthesis, can be targeted by the synthetic compound brequinar to suppress the viability and teratoma-forming capability of mouse PSCs (both ESCs and iPSCs) in NOD/SCID mice without significant adverse effects. Additionally, treatment with brequinar significantly reduced the number of Nanog-positive pluripotent cells compared to Nestin-positive neural cells in co-cultures of mouse PSCs and neural stem cells. These findings suggest that the proliferation and survival of mouse PSCs depend on the pyrimidine biosynthesis pathway (Kondo, 2021). However, the effect of brequinar on hPSCs was not analyzed in this study, leaving the potential application of DHODH inhibitors in hPSC-based cell therapy unproven.

Chromatin topology appears to play a crucial role in maintaining the stemness and pluripotency of PSCs (Pelham-Webb et al., 2020; Platania and Sexton, 2020). Recently, Chour et al. demonstrated that the chemotherapeutic drug doxorubicin can effectively and selectively eliminate undifferentiated hESCs in culture without causing cardiotoxicity in hESC-derived cardiomyocytes when used at a low dose of 0.01 μmol/L (Chour et al., 2021). Doxorubicin inhibits topoisomerase II, which is essential for DNA replication and for maintaining proper chromosome condensation and segregation (Kciuk et al., 2023). To determine if pretreatment of hESC with a minimal dose of doxorubicin could prevent teratoma formation *in vivo*, 5 × 10^5^ hESCs were exposed to 0.01 μmol/L of the drug for 48 h. These cells were then mixed with 1 × 10^6^ hESC-cardiomyocytes and transplanted subcutaneously into immunodeficient NOD/SCID mice. The experiment showed that teratomas formed only in mice receiving hESC-cardiomyocytes mixed with untreated hESCs but not in those mixed with doxorubicin-pretreated hESCs. However, this experimental design does not fully mimic the real-world scenarios, where undifferentiated hPSCs would be exposed to the drug in a mixture with the differentiated cell population from which they need to be removed. Although the authors demonstrated that doxorubicin does not exert cytotoxic effects on hESC-cardiomyocytes *in vitro*, its potential to prevent teratoma formation *in vivo* without compromising the viability and therapeutic efficacy of transplanted cardiomyocytes remains to be established.

Etoposide, a chemotherapeutic agent that inhibits topoisomerase II and induces DNA damage, has also shown promise in selectively eliminating contaminating murine ESCs and iPSCs. Smith et al. demonstrated that murine PSCs are significantly more sensitive to apoptosis induced by etoposide than their differentiated derivatives, due to increased levels of the pro-apoptotic protein p53 upregulated modulator of apoptosis, also known as Bcl-2-binding component 3 (Smith et al., 2012). Pre-treatment of spontaneously differentiated progeny of murine PSCs with etoposide significantly reduced tumor volume or completely abolished tumor formation upon subcutaneous transplantation into nude mice. In subsequent experiments using murine iPSC-derived cardiac progenitor cells, it was shown that etoposide significantly decreases teratoma formation after injection into infarcted murine hearts (Wyles et al., 2014). Later, this research group developed an etoposide sensitivity assay to assess the quality of hiPSC lines, based on the observation that the half-maximal effective concentration for 115 individual hiPSC lines after 24 h of etoposide exposure was less than 300 nM, correlating with high-quality hiPSC clones (Secreto et al., 2017). However, there is still no experimental evidence confirming that etoposide can decrease the risk of teratoma formation from contaminating hPSCs without being toxic to their differentiated progeny, such as cardiac or neural cells.

Finally, the cyclin-dependent kinases inhibitor dinaciclib, which induces DNA damage, upregulates the p53 protein levels, and decreases levels of the survival factor MCL-1 (myeloid leukemia cell differentiation protein) in hiPSCs, has been shown to induce apoptotic cell death in undifferentiated hiPSCs without significantly affecting hiPSC-derived cardiomyocytes (Alsayegh et al., 2017). Thus, substances that damage DNA or disrupt its function and topology more effectively in undifferentiated hPSCs than in their derivatives appear to be promising candidates for eliminating residual iPSCs from differentiated cells intended for use in regenerative medicine.

### Other small-molecule compounds

As mentioned earlier, some natural compounds and nutritional supplements, such as the flavonoid quercetin, exhibit selective cytotoxicity toward undifferentiated hPSCs (Lee et al., 2013c). Go et al. conducted a screening of various in-house flavonoids and found that luteolin was even more potent than quercetin in effectively removing hPSCs. They demonstrated that luteolin promoted hPSC removal via activation of the p53 pathway, without adversely affecting hESC-derived cardiomyocytes (Go et al., 2020). Since cardiomyocytes are difficult to purify due to their lack of unique surface markers, strategies that eliminate undifferentiated hPSCs through alternative mechanisms could help reduce the risk of teratoma formation, thereby enhancing the safety of hPSC-derived cardiac cell therapy.

Kim et al. suggested that the phenolic compound caffeic acid, found in plants, exhibits PSC-toxic effects in a co-culture system consisting of hPSCs and human dermal fibroblasts or hiPSC-derived MSCs. Caffeic acid was also shown to prevent teratoma formation from hPSCs (Kim et al., 2022), representing another natural compound with the ability to target undifferentiated hPSCs. A recent study also demonstrated that cardiac glycosides, such as naturally occurring compounds extracted from *Digitalis lanata* digoxin and lanatoside C, are toxic to hESCs but not to human bone marrow-derived MSCs, hESC-derived MSCs, neurons, or endothelial cells and are capable of preventing teratoma formation (Lin et al., 2017). Like other FDA-approved drugs mentioned earlier, cardiac glycosides are already in clinical use and could be repurposed for hPSC-based regenerative medicine without major regulatory hurdles.

Burkert and coworkers reported that both human and murine PSCs, but not cardiomyocytes derived from them, are vulnerable to compounds in the salicylic diamine group (Burkert et al., 2021). These compounds were selected because previous studies found that they could selectively induce apoptosis in leukemia and lymphoma cells (Gao et al., 2007). Treatment of mouse ESC- and iPSC-derived embryoid bodies in suspension differentiation cultures with the most effective salicylic diamine, which the authors named as small molecule 6 (SM6), significantly reduced the number of contaminating iPSCs without causing lasting adverse effects on iPSC-derived cardiomyocytes. Analysis of mitochondrial respiration in murine and hiPSCs revealed that SM6 inhibited the oxygen consumption rate in these cells, whereas in cardiomyocytes it exhibited no or only minor and reversible effects on oxygen consumption, sarcomeric integrity, DNA stability, apoptotic rate, ROS levels, or beating frequency. Treatment of one million murine iPSC-derived cardiomyocytes, containing less than 0.001% residual undifferentiated iPSCs, with 10 μM of SM6 completely abolished teratoma formation in two out of seven immunodeficient NSG mice after intramuscular injection. The remaining five animals developed teratomas but with a significant delay of several weeks compared to those injected with untreated cells (Burkert et al., 2021). These findings suggest that further optimization of the treatment protocol and the development of more potent SM6 derivatives are necessary to enhance the anti-teratogenic potency of these drugs.

Another hPSC-killing agent is the small molecule WX8, along with other inhibitors of the phosphatidylinositol kinase PIKfyve. These chemicals, previously shown to induce cell death in cancer cells (Ikonomov et al., 2019), were recently found to selectively promote programmed cell death in hESCs, hiPSCs, and human ECCs by disrupting lysosome homeostasis and autophagy. WX8 reduced the rate of teratocarcinoma formation from injected human ECCs and inhibited the proliferation of undifferentiated Ki67-, OCT4-, and SOX2-positive PSCs within the resulting teratocarcinomas (Chakraborty et al., 2022). However, a concern with this study is that PIKfyve inhibitors also decreased proliferation in both undifferentiated hPSCs and differentiated cells, albeit at a lower rate in the latter. This raises the need for further investigation to determine whether these small molecules adversely affect the normal growth and functionality of hPSC-derived differentiated cells. Moreover, the fact that not all tumors were prevented by inhibitor treatment suggests that residual undifferentiated hPSCs may still pose a tumorigenic risk in cell therapy.

Finally, Cho et al. reported that the application of the hPSC-specific fluorescent probe CDy1 selectively triggered apoptosis in hPSCs when exposed to visible light, while endothelial cells grew normally under the same treatment conditions. CDy1 can be used for hPSC isolation without harming the viability or functionality of the cells. Its toxicity is activated upon illumination, leading to the production of mitochondrial ROS and the induction of apoptotic death. Importantly, the treatment of undifferentiated hPSCs with CDy1 reduced teratoma formation in mice (Cho et al., 2016). However, the results of this study cannot be generalized to other differentiated cell types, as the selectivity and efficiency of this probe have only been experimentally demonstrated for endothelial cells contaminated with hPSCs.

Compared to antibodies, proteins, and other PSC-inhibitory strategies, small molecules offer several advantages: (1) they are more accessible and easier to use than other reagents, (2) they can penetrate cells more easily due to their small size, (3) they can be produced and applied at a much lower cost, (4) they are easily adaptable to large-scale cell production processes in bioreactors, (5) they offer flexibility in combinatorial application at specific times, durations, and doses, and (6) FDA-approved hPSC-toxic drugs could even be administered to patients to treat tumors if they occur at any time after transplantation.

## Other strategies

### Specific hPSC killing by activated immune cells

Another strategy for specific hiPSC elimination involves using cytotoxic T lymphocytes (CTLs) that target hPSC-specific antigenic peptides presented by human leukocyte antigen class I molecules on the cell surface. Okada and colleagues identified glypican-3 (GPC3) as a hiPSC-specific antigen and demonstrated that GPC3-reactive CTLs could selectively kill undifferentiated hiPSCs *in vitro*. Moreover, this approach effectively reduced teratomas formed in immunodeficient mice within 3 weeks (Okada et al., 2019).

### Intracellular peptide assemblies

Alkaline phosphatase (ALP) is a prominent marker of hPSCs, catalyzing the dephosphorylation of phosphopentapeptides. Liu and colleagues utilized L-phosphopentapeptide, which becomes dephosphorylated by ALP, leading to the formation of intranuclear peptide assemblies composed of α helices and aggregated strands. Incubation of hiPSCs with L-phosphopentapeptide, consisting of four L-leucine residues and a C-terminal L-phosphotyrosine, selectively eliminated hiPSCs in a mixed cell population containing hiPSCs and non-hiPSCs, such as HEK293 and hematopoietic progenitor cells (Liu et al., 2021).

### Modifying the cell culture medium

Cell behavior in response to different culture media can be exploited to detect and separate various types of cells. A well-known example is the separation of human MSCs from the bulk of hematopoietic cells using plastic tissue culture plates, as most hematopoietic cells are non-adherent and can be easily removed during culture refeeding (Lennon and Caplan, 2006). Additionally, culture media with high lactate levels and no glucose have been reported to support the growth of cardiomyocytes but not hPSCs, from which the cardiomyocytes were derived (Tohyama et al., 2013). Similarly, a culture medium lacking glucose and arginine selectively eliminated hiPSCs in a co-culture system with hepatocytes (Tomizawa et al., 2013). Furthermore, co-culturing hPSCs with retinal pigment epithelial (RPE) cells selectively induced hPSC death, attributed to the secretion of pigmented epithelium-derived factor (PEDF) by RPE cells—a 50 kDa glycoprotein also expressed in various other tissue types (Cai et al., 2006; Chuderland et al., 2014; Karakousis et al., 2001; Matsuoka et al., 2004). PEDF has also shown anti-tumor properties in nerve cells, and its use inhibited hPSC growth *in vitro*. Remarkably, tumor formation decreased significantly when hiPSCs were co-transplanted with an hiPSC-derived RPE sheet into NOG mice subcutaneously during a 30-week follow-up (Kanemura et al., 2013). However, a limitation of this approach is the lack of knowledge regarding PEDF’s potential effects on the fate and function of differentiated cells, such as chondrocytes, where PEDF plays important roles in endochondral ossification by promoting matrix degradation and suppressing cartilage-specific gene expression (Klinger et al., 2017), in addition to RPE cells. Although the culture media-based approach is straightforward to apply, it is not broadly applicable to all differentiated cell types.

### Irradiation for eradication

hPSCs exhibit an innate sensitivity to radiation due to impaired DNA repair mechanisms and a natural inclination toward apoptosis (Desmarais et al., 2012; Filion et al., 2009). For instance, when hiPSC-derived cardiomyocytes were irradiated with X-rays, the expression of pluripotency genes such as *LIN28A*, *NANOG*, *OCT3/4*, and *SOX2* in the hiPSC-derived cardiomyocyte population was markedly reduced, while no significant changes were observed in the typical function and features of cardiomyocytes (Takeda et al., 2021). Although this finding suggests that X-ray treatment may enhance the safety of transplanted hiPSC-derived cardiomyocytes, it also raises concerns about potential genomic damage to the differentiated cells, which could compromise their clinical efficacy and safety.

### Bee venom

Kim and colleagues discovered that bee venom causes membrane breakdown and focal adhesion loss, leading to cell death in hiPSCs. Moreover, bee venom inhibited the *in ovo* growth of teratomas formed by hiPSCs, without exhibiting cytotoxic or genotoxic effects on differentiated white and brown adipocytes derived from hiPSCs, which have a fibroblast-like morphology (Kim et al., 2020a). However, it remains unclear whether bee venom can also inhibit teratoma formation in more established animal models and whether it is truly non-cytotoxic to various differentiated cells.

### Traditional medicine

Traditional medicine offers promising approaches to treating various diseases (Li and Weng, 2017; Rizvi et al., 2022). The ethanol extract of *Magnolia officinalis* (Magnolia cortex) has been found to exert cytotoxic effects on hiPSCs and inhibit their *in ovo* tumor formation by inducing apoptosis, without negatively impacting the growth of differentiated cells, such as human dermal fibroblasts (Kim et al., 2020b). In another study, researchers investigated the effect of the spike extract of *Prunella vulgaris* L. (Spica Prunellae), a traditional medicinal herb known for its anti-cancer and antioxidant effects, on hiPSC viability and tumorigenicity. They reported that the ethanol extract of this plant suppressed the growth of undifferentiated hiPSCs by inducing G2/M cell-cycle arrest, activating caspases, and increasing ROS production. Since the ethanol extract of Spica Prunellae was unable to eliminate *p53*-knockout hiPSCs, it was suggested that its anti-hPSC effects are mediated through the *p53* pathway. Notably, *p53*-knockout hiPSCs generated teratomas *in ovo*, whereas wild-type hiPSCs did not form teratomas (Kim et al., 2020c). Further research is needed to identify the active ingredient(s) responsible for these medicinal herbs’ effects on hPSC growth and tumorigenesis.

## Methods to analyze successful PSC removal

**Figure 3:**
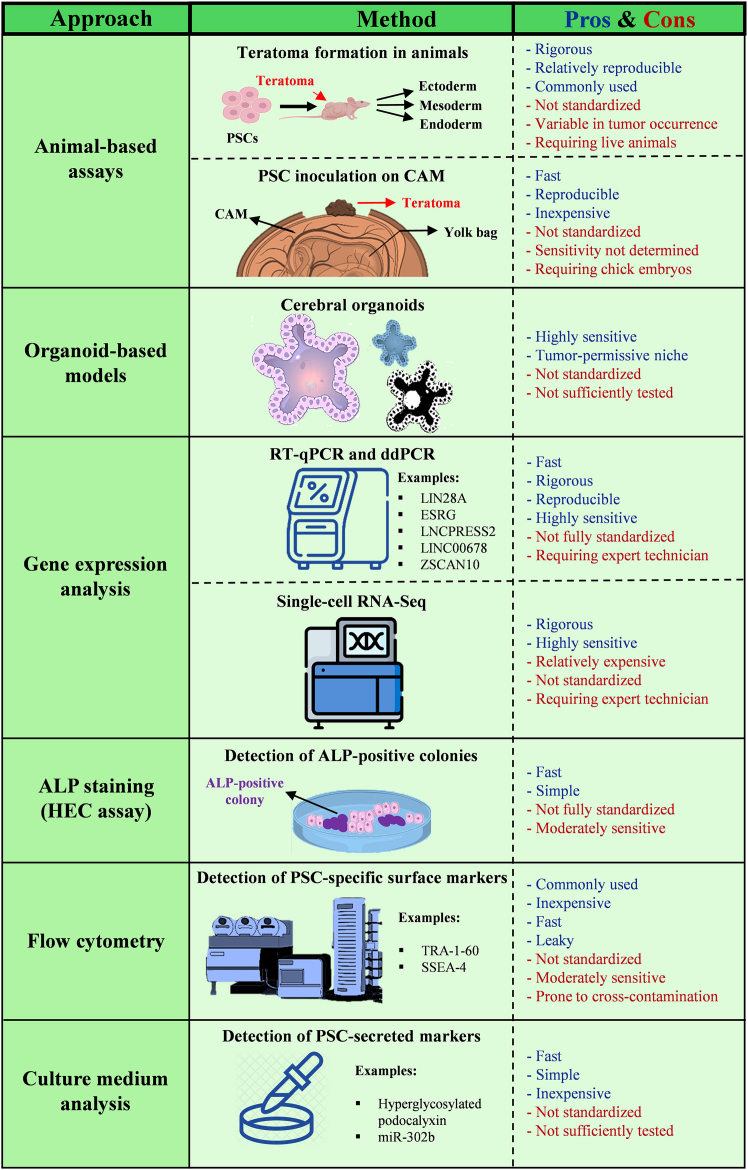
Various approaches to the detection of undifferentiated PSCs in clinical settings This figure summarizes different methods for detecting residual undifferentiated PSCs in clinical-grade cell products. Animal-based assays, such as teratoma formation and CAM-based tumorigenesis assay, vary in sensitivity and practicality. Organoid models, like cerebral organoids, offer high sensitivity but require further validation. PCR-based techniques, including RT-qPCR and ddPCR, enable ultra-sensitive detection of trace PSC contamination. Additional approaches include ALP staining of undifferentiated PSCs in a so-called highly efficient culture (HEC) assay, flow cytometry for PSC surface markers, culture medium analysis for secreted markers, and scRNA-seq, each with distinct advantages and limitations in sensitivity, cost, and scalability.

**Figure 4:**
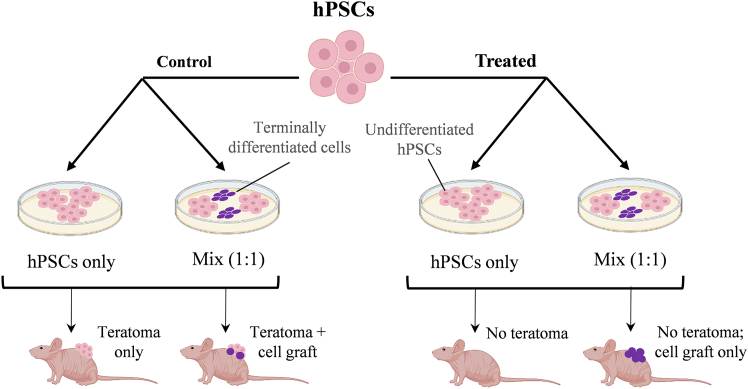
Teratoma formation by PSCs in nude mice, with and without anti-PSC agents Although treatment reduces the number of viable PSCs, the same quantity of PSCs should be used for both control and treatment groups during each injection. To prepare samples for teratoma formation analysis, undifferentiated PSCs are mixed with differentiated cells at different ratios, such as 1:1, for injection. To simulate a more realistic clinical scenario, the ratio of PSC-derived differentiated cells to undifferentiated cells should be increased significantly to better assess the efficiency of PSC removal and the risk of tumor formation after cell transplantation in this assay.

To validate the effective removal of undifferentiated PSCs from differentiated cell populations, various methods have been employed. This quality control step is critical in determining the efficiency and selectivity of PSC elimination by a given technique. Figure 3 summarizes various methods for detecting residual undifferentiated PSCs in differentiated cell cultures. While these analyses primarily focus on studying PSC-toxic chemical compounds to maximize the safety of PSC-based cell therapies, they can also be applied to other studies involving PSCs to examine whether a particular treatment affects PSC behavior in their undifferentiated and/or differentiated states. Since PSCs produce teratomas when injected into immunocompromised animals, the teratoma formation assay is one of the most rigorous and most commonly used methods to evaluate the safety of PSC products after a given PSC elimination strategy has been applied (Montilla-Rojo et al., 2023) (Figure 4). However, the disadvantage of this test is that it lacks standardization, exhibits high inter-cell-line variation in tumor incidence and formation latency, requires live animals, and involves long observation periods to obtain results (Yasuda et al., 2018). In addition, for hPSC derivatives, this method may not be sensitive enough to detect very low levels of hPSC contamination, overlooking the risk these cells may pose in the clinic. An alternative *in vivo* model to assess the pluripotency of hiPSCs is based on the inoculation of hiPSCs on the chicken egg chorioallantoic membrane (CAM) (Weber et al., 2021). In this method, seeding two or four million hiPSCs on CAM resulted in teratoma formation in 70% or 100% of eggs, respectively, within only 9 days. This simple, fast, inexpensive, and reproducible method has been used to demonstrate that treating hPSCs with various plant extracts limits teratoma formation (Kim et al., 2020a, 2020b, 2023, 2020c). However, the sensitivity of this assay has not yet been determined.

As a potential *in vitro* alternative to animal-based tumorigenicity assays, cerebral organoids have recently demonstrated superior sensitivity for detecting residual hPSCs. These models recapitulate a tumor-permissive microenvironment that enhances the proliferative capacity of spiked hPSCs, outperforming both standard cerebral organoids and *in vivo* mouse models in detection efficiency while supporting functional maturation of therapeutic cell products (Xue et al., 2024).

In addition to *in vivo* methods, various animal-free methods have been developed in recent years to assess the presence of undifferentiated PSCs in a differentiated cell product (Watanabe et al., 2023). They include quantitative reverse-transcription PCR (RT-qPCR) or droplet digital PCR (ddPCR) for determining the expression of pluripotency markers such as *LIN28A* and *ESRG* or long non-coding RNAs (lncRNAs) *LNCPRESS2*, *LINC00678*, and *LOC105370482* in iPSC-derived cells (Kuroda et al., 2012, 2015; Lemmens et al., 2023; Wu et al., 2023; Yasuda et al., 2024), detection of ALP-positive hiPSC colonies spiked in cultures of hiPSC-derived cardiomyocytes (Watanabe et al., 2023), flow cytometry for identifying PSC-associated surface markers (e.g., TRA-1-60 and SSEA-4) (Kuroda et al., 2012), and detection of hPSC-specific markers released into culture media such as hyperglycosylated podocalyxin (Tateno et al., 2014) or miR-302b (Masumoto et al., 2022). In addition, single-cell RNA sequencing (scRNA-seq) represents a suitable method to detect and quantify residual PSCs in therapeutic cell products (Camp et al., 2018). However, scRNA-seq is relatively expensive, and capturing extremely rare PSCs requires very high cell numbers. Finally, digital soft agar colony formation assay can be used for detecting malignantly transformed cells in therapeutic cell products. However, while this assay was capable of detecting 0.0001% HeLa cells spiked into human MSCs (Bando et al., 2024), it was unable to detect hiPSCs even in the presence of a ROCK inhibitor that supports the survival of dissociated hPSCs (Kuroda et al., 2012). The limit of PSC detection varies widely between different methods. Flow cytometry using the anti-TRA-1-60 antibody was able to detect 0.1% undifferentiated hiPSCs that were spiked in primary RPE cells, while *Lin28*-RT-qPCR could detect an equivalent of a single hiPSC in 50,000 RPE cells. The ddPCR assay targeting *ESRG* and *LIN28A* genes could detect up to 0.0003% hPSC impurities in hiPSC-derived cardiomyocytes (Yasuda et al., 2024), while ddPCR for *LNCPRESS2* and *LINC00678* lncRNAs could detect one hiPSC in one million islet cells (Wu et al., 2023). Furthermore, the ddPCR method specific for *ESRG* and *ZSCAN10* detected a trace of undifferentiated hiPSCs to a spiked level of 0.0001% (Shi et al., 2022). Regulatory agencies such as the FDA and EMA often recommend a combination of different methods to ensure the safety and efficacy of hPSC-derived therapies (Sato et al., 2019).

## Conclusion and perspective: What lies ahead?

As the clinical use of hPSC-derivatives for treating specific diseases is expected to increase in the coming years, the safety issues associated with these cells will become increasingly important. Since contamination with only a few undifferentiated hPSCs could lead to teratoma formation upon injection of hPSC-derived cells, it is crucial to develop effective strategies for removing undifferentiated, tumorigenic hPSCs and malignantly transformed iPSC derivatives from hPSC-derived cell therapy products before transplantation into patients.

Although various strategies have been proposed for the removal of PSCs, not all methods are equally suitable for clinical application. Methods involving stable genetic modification of cells are unlikely to be favored due to their labor-intensive nature, time requirements, and potential failure to pass regulatory approval. Introducing foreign sequences into the genome may itself pose tumorigenic and immunogenic risks. RNA interference (RNAi) technology appears to hold greater potential in specifically targeting hPSCs and preventing teratoma formation because it requires only the transient expression of synthetic RNA molecules, such as miRNAs or siRNAs, in target cells. However, the stability of these agents might be suboptimal, their synthesis could be challenging, and the efficiency of delivery into target cells might be compromised, especially in large-scale production processes, thus reducing their hPSC-eliminating potency. On the other hand, small molecules seem to hold great potential in this context due to their lower cost, simplicity, accessibility, scalability, and flexibility in combinatorial application at specific times, durations, and doses. This represents an advantage over other approaches. Moreover, some hPSC-toxic agents, such as cardiac glycosides, metformin, doxorubicin, and atorvastatin, are FDA-approved drugs, or they are in use as nutritional supplements, such as quercetin, making them particularly attractive candidates for hPSC elimination in regenerative medicine applications. Furthermore, in cases where a teratoma has already formed in the patient’s body following hPSC-based cell transplantation, FDA-approved hPSC-toxic small molecules may be more practical than unapproved ones. However, they may not be effective under such circumstances because most cells forming teratomas are differentiated and therefore may not be sensitive to these drugs. Interestingly, the immunohistological analysis of the immature teratoma that was formed in a patient receiving PSC derivatives for diabetes treatment revealed that this tumor contained OCT4- and SOX2-positive cells (Han et al., 2022), suggesting that the presence of undifferentiated hPSCs in teratomas may depend on their pathological grade as well as the host in which they develop. Addressing this challenge will require discovering novel agents capable of shrinking pre-existing teratomas *in vivo*.

Despite the advantages of small molecules, any chemical substance may carry the risk of unwanted side effects on some types of differentiated cells, potentially limiting its widespread use. Therefore, the best strategy to maximize the safety of hPSC-based therapies will probably be in choosing a method or combination of methods capable of completely eliminating all hPSCs before the differentiated cells are transplanted into patients, thereby reducing the risk of teratoma formation as much as possible. To our knowledge, no studies have experimentally tested whether combining different approaches is more effective in eliminating hPSCs while preserving the viability and functionality of the differentiated cells compared to using a single approach. One approach that could be easily combined with small molecules is the use of antibody-drug conjugates, some of which are FDA approved (such as brentuximab vedotin), or directly cytotoxic antibodies that can kill hPSCs within minutes (such as antibodies against PODXL or *O*-linked glycan epitope). Although producing these biological reagents is more complex and expensive compared to chemical molecules, they can be manufactured in large quantities, and their use is simple and compatible with bioreactor-based differentiation processes required to produce large quantities of cells needed for clinical applications. Given the importance of manufacturing conditions for future hPSC-based cell therapies, it is critical to discover and characterize agents capable of removing contaminating hPSCs under these conditions.

It is tempting to speculate that in the near future, the need for specialized hPSC-removal strategies in producing hPSC derivatives may be significantly reduced or even eliminated, as many types of differentiated cells can now be generated from hPSCs with high yield and purity under conditions not conducive to the survival and growth of undifferentiated hPSCs. However, most of these protocols have only been validated in small-scale differentiation protocols, so the question remains whether cells produced in large-scale bioreactor-based processes might still contain contaminated hPSCs at very low levels that escape reliable detection. Since even a single case of teratoma occurrence in the clinic after transplantation of hPSC-based therapeutics could be highly detrimental to the entire stem cell field, concerns of hPSC-mediated teratogenesis are well justified. It is therefore highly likely that methods to eradicate hPSCs from their differentiated derivatives will be routinely employed as a safety measure to eliminate the tumorigenic risk of these cellular products. In order to fulfill their role, it is crucial that these methods are highly selective and potent, that their production is straightforward and affordable, and that their application is simple.

Moreover, even in the complete absence of undifferentiated hPSCs, transplanted hPSC derivatives could potentially lead to tumors other than teratomas due to malignant transformation or simply due to proliferation of the transplanted cells or their less mature progenitors. Addressing this problem may require strategies other than those described earlier for hPSC removal. The risk of malignant transformation of the transplanted cells can be minimized through strict quality control of their genetic integrity and careful evaluation of the *in vivo* proliferative behavior of non-hPSC contaminants in final hPSC-based therapeutic products. The aforementioned fail-safe strategies cover this aspect but require genetic modification.

In conclusion, employing strategies to completely remove undifferentiated hPSCs upon hPSC-derived cell injection is crucial to ensure the safety of cell therapies. Protein- and antibody-based strategies may not be the best options as they provide only partial hPSC removal. Small molecules appear to hold the greatest potential for effectively eradicating hPSCs, provided that they are non-genotoxic and do not adversely affect the functionality of the hPSC-differentiated cells. Moreover, hPSC-toxic agents should ideally have good safety profiles in humans so that if a tumor forms from hPSC-based cell therapies, they can be used to either shrink or suppress the further growth of the formed tumors. The safety of the anti-hPSC agents is of utmost importance since an agent intended to ensure the safety of cell therapy must not cause serious side effects, especially when a tumor formed from hPSC-based cell therapy in a patient. Finally, genetic approaches based on the stable modification of the genome of hPSCs could still be considered, especially if the expressed transgenes are controlled by regulatory circuits that will be activated only in tumor but not healthy transplanted cells. Recent advances in targeted transgene integration using CRISPR-Cas-based technologies may allow for the consideration of suicide gene strategies.

## Acknowledgments

We would like to thank Martin Pera from the Jackson Laboratory for critical review of the manuscript. S.M. was funded by 10.13039/501100012155National Institute for Medical Research Development (NIMAD) Grant (973138). P.S. was supported by funds from the 10.13039/501100002915Fondation pour la Recherche Médicale (DEQ20170336757), the LabEx REVIVE (ANR-10-LABX-73), and the LabEx “DEVweCAN” (ANR-10-LABX-0061). T.Š. was supported by funds from the Ministry of Culture and Science of the State of North Rhine-Westphalia (005-2305-0041) and the Köln-Fortune Program (390/2021).

## Author contributions

S.M. conceptualized and designed the study. R.B., A.Y.M., and S.M. drafted the manuscript and created the figures. T.Š. wrote and critically revised the manuscript and modified the figures. P.S. critically revised the manuscript. All authors read and approved the final version of the manuscript for submission.

## Declaration of interests

The authors declare no competing interests.
